# Supplementary material for: Splice-Junction-Based Mapping of Alternative Isoforms in the Human Proteome
Source: Cell Rep. Author manuscript; Available in PMC 2020 Jan 15. (PMC6961840; doi:10.1016/j.celrep.2019.11.026)
Supplement: 2 [file NIHMS1546469-supplement-2.zip › DF1/PXD009737/TestisLysC/TestisLysC_3_USP13_ASDIDESSVMQLAEMGFPLEACRK.pdf]

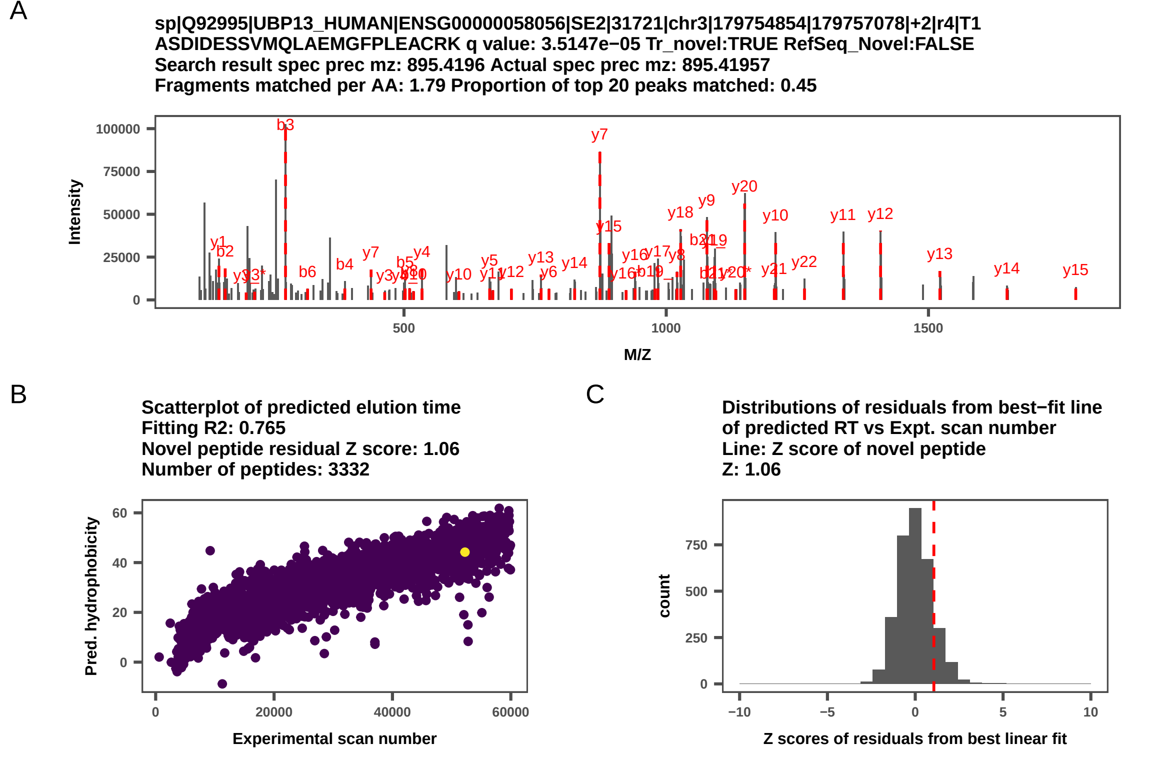

A
sp|Q92995|UBP13_HUMAN|ENSG00000058056|SE2|31721|chr3|179754854|179757078|+2|r4|T1
ASDIDESSVMQLAEMGFPLEACRK q value: 3.5147e−05 Tr_novel:TRUE RefSeq_Novel:FALSE
Search result spec prec mz: 895.4196 Actual spec prec mz: 895.41957
Fragments matched per AA: 1.79 Proportion of top 20 peaks matched: 0.45
Intensity
100000
75000
50000
25000
0
500
1000
1500
M/Z
B
Scatterplot of predicted elution time
Fitting R2: 0.765
Novel peptide residual Z score: 1.06
Number of peptides: 3332
Pred. hydrophobicity
60
40
20
0
0
20000
40000
60000
Experimental scan number
C
Distributions of residuals from best−fit line
of predicted RT vs Expt. scan number
Line: Z score of novel peptide
Z: 1.06
count
750
500
250
0
−10
−5
0
5
10
Z scores of residuals from best linear fit
